# External apical root resorption after orthodontic treatment: Incidence, severity and risk factors

**DOI:** 10.34172/joddd.2021.017

**Published:** 2021-05-05

**Authors:** Fatih Bayir, Esra Bolat Gumus

**Affiliations:** ^1^Orthodontist; Private Practice, Antalya, Turkey; ^2^Department of Orthodontics, Faculty of Dentistry, Akdeniz University, Antalya, Turkey

**Keywords:** Orthodontic treatment, Risk factors, Root resorption

## Abstract

**Background.** This study aimed to evaluate the incidence and severity of orthodontically induced inflammatory external apical root resorption (OIIEARR) and the relationship between OIIEARR and possible risk factors such as orthodontic treatment type, treatment duration, gender, and age of the patients. A further aim was to determine the prevalence of OIIEARR in different tooth groups.

**Methods.** The study sample consisted of 1356 orthodontically treated patients (857 females and 498 males; mean age: 14.4±2.8 years). OIIEARR was evaluated using pre- and post-treatment panoramic radiographs for all the tooth groups. Teeth with severe resorption were also assessed. Patient- and treatment-related risk factors for OIIEARR were assessed statistically using Pearson’s chi-squared test, independent-samples *t* test, and one-way ANOVA.

**Results.** The incidence of severe root resorption following orthodontic treatment was 14.8%. Males exhibited a higher incidence of root resorption compared to females. Orthodontic treatment duration and treatment with extractions were positively correlated with OIIEARR (*P* < 0.05). OIIEARR was observed most frequently in maxillary incisors, followed by mandibular incisors.

**Conclusion.** Orthodontic treatment with extraction, prolonged treatment duration, and large movements of the incisors should especially be taken into consideration for OIIEARR risk. Routine radiographic follow-up during orthodontic treatment is recommended.

## Introduction

Orthodontics is probably the only dental specialty that uses the inflammatory process to treat functional and aesthetic problems.^1^ This inflammatory process, which is the fundamental component behind the root resorption process, is essential for orthodontic tooth movement.^1^ Orthodontically induced inflammatory external apical root resorption (OIIEARR) is an undesirable but unavoidable pathological consequence of orthodontic tooth movement. Three degrees of OIIEARR are reported in the literature: cemental or surface resorption with remodeling, dentinal resorption with repair, and circumferential apical root resorption with root shortening as evidence.^2^ Although external apical root resorption related to orthodontic treatment is rarely serious, it is a devastating event when it is radiographically recognized.

The extent of the root resorption inflammatory process depends on many factors, such as the aggressiveness of the various resorbing cells and the vulnerability and sensitivity of the tissues involved. Individual variations and susceptibility, which are related to this process, remain beyond our understanding.^2^ Factors such as bone density and morphology, the shape of the roots, previous trauma,^3^ the type of malocclusion, pre-treatment patient age,^4^ patient gender,^5^ the duration of active treatment,^3,6,7^ orthodontic mechanics and the magnitude of force^8^ and orthodontic treatment type, with or without extraction^9^ have been reported as significant for the occurrence of OIIEARR. However, we are still unable to predict the incidence and extent of OIIEARR after orthodontic force application. An assessment of the incidence and risk factors of OIIEARR would clinicians with treatment planning and could help avoid potential biological damage and legal implications.

This retrospective study analyzed, using pre-treatment and post-treatment panoramic radiographs, the incidence and degree of OIIEARR, the potential risk factors related to the patient or treatment and the degree of OIIEARR in different tooth groups. To our knowledge, this study has the largest sample size reported in this type of study.^9-13^

## Methods

### Sample selection

The study material was selected from the archives of the Akdeniz University, Faculty of Dentistry, Department of Orthodontics. In total, 1678 files of patients who were treated between 2012 and 2019 were analyzed, and the pre- and post-treatment panoramic radiographs of 1356 patients (857 females and 498 male; mean age: 14.4±2.8 years) who met the following inclusion criteria were used: anamnesis, treatment planning, and clinical procedure sheets properly filled in; permanent dentition or at least one of the molar or incisor teeth with complete root formation; no history of previous orthodontic treatment or dental trauma; no craniofacial anomalies, systemic disorders (such as chronic asthma, thyroid dysfunction, etc.) or parafunctional habits (bruxism, tongue thrusting, etc.). Conventional edgewise appliances were used for all fixed orthodontic treatments.

Panoramic radiographs of 211 patients in whom the roots were distorted or not clearly visualized with low image quality and eight patients with pre-treatment root resorption were excluded from the study. Another 23 patient radiographs were not included in the study due to the absence of previously mentioned information. Only teeth with complete root formation were examined, and teeth with periapical lesions or endodontic treatment were excluded.

Table 1 presents the age and gender distributions of the patients.

**Table 1:** Number and percentage of patients exhibiting post-treatment OIIEARR

	**Orthodontically induced inflammatory external apical root resorption (OIIEARR)**									
	**None (degree 0)**		**Resorption (degrees 1-2-3)**		***P*** ** value**	**None to mild resorption (degree 0-1)**		**Severe resorption** **(degree 2-3)**		***P*** ** value**
	**No.**	**%**	**No.**	**%**		**No.**	**%**	**No.**	**%**	
Male	337	67.7	161	32.3	0.002*	430	86.3	68	13.7	0.2
Female	644	75	214	25		725	84.5	133	15.5	
Total	981	72.3	375	27.7		1155	85.2	201	14.8	

*P < 0.05, Chi-square test.

### OIIEARR measurements

**Figure 1:**
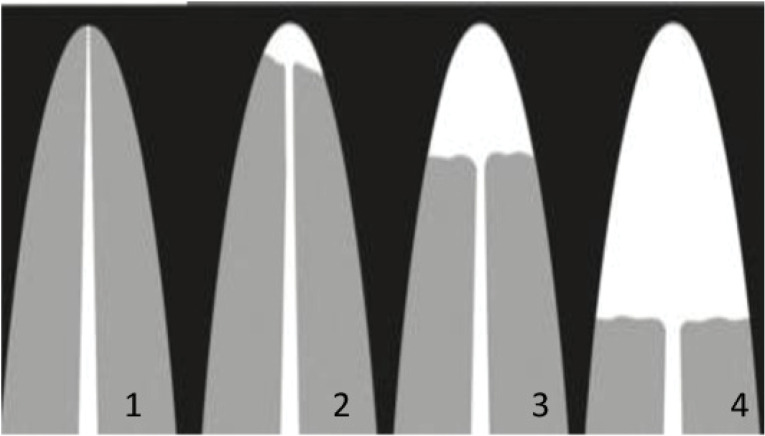
Degrees of external apical root resorption. Degree 0: no resorption; Degree 1: resorption of up to 2 mm of the root length; Degree 2: resorption from 2 mm up to 1/3 of the root length; Degree 3: severe root resorption, > 1/3 of the root length.

Panoramic radiographs were obtained using the same Planmeca ProMax panoramic device, following the manufacturer’s instructions in a standard manner. All the radiographs were evaluated using the same LED monitor by the same investigator (F.B.). After four weeks, all the measurements of 120 randomly selected patients were repeated, and inter-observer variability was assessed. The post-treatment root lengths of all the teeth were compared with the root lengths on the pre-treatment panoramic radiographs. The index suggested by Malmgren et al^14^ and modified by Sharpe et al^15^ was accepted as a visual qualitative method used to assess the degree of OIIEARR due to its broad acceptance and applicability (Figure 1).

- Degree 0: the absence of resorption
- Degree 1: resorption of up to 2 mm of the root length
- Degree 2: resorption from 2 mm up to 1/3 of the root length
- Degree 3: severe root resorption, > 1/3 of the root length

Post-treatment panoramic radiographs of 1356 patients with no root resorption in the examined pre-treatment radiographs and all the permanent teeth between #16 and #46 were evaluated and classified based on their resorption degrees. The teeth exhibiting degrees 0 and 1 were considered as ‘none to mild resorption,’ and teeth exhibiting degrees 2 and 3 were considered as ‘severe resorption’ concerning the clinical significance of root resorption.

In order to determine potential risk factors, the following variables were assessed: the age at the beginning of treatment, gender, treatment type (the use of active removable appliances before fixed appliances or not, with or without extraction), and treatment duration (with removable appliances, with fixed appliances, and total treatment duration). A further evaluation for treatment type and treatment duration distributions according to patient gender was performed, and the degree of OIIEARR in different dental arches (maxilla and mandible) and tooth groups (incisors, premolars, etc.) were examined.

### Statistical analysis

All the statistical analyses were performed using SPSS 23.0 (SPSS for Windows, version 23.0; Chicago, IL). In-class correlation coefficients were calculated to assess inter-observer variability with Cronbach’s alpha. The gender distribution of the patients was assessed by frequency analysis. Pearson’s chi-squared test was used to determine the OIIEARR-gender and OIRR-treatment type relationships. Independent-samples t-tests were applied to analyze the means and distributions of the pre-treatment ages of the patients and the distribution of OIIEARR in different dental arches and tooth groups. The relationships between OIIEARR and total treatment duration and between treatment duration and fixed appliances were analyzed by one-way ANOVA. All the values were considered significant at *P* < 0.05.

## Results

Inter-observer correlation coefficients were between 0.915 and 0.945, with almost 100% agreement. Pre-treatment resorption with different degrees was observed in only eight of 1678 patients (0.47%), who were excluded from the study.

Apical root resorption was observed in 375 of 1356 patients’ post-treatment radiographs (27.7%), and 201 patients (14.8%) developed severe OIIEARR (degree 2‒3). Males developed OIIEARR degrees 1‒3, significantly more frequent (n=161, 32.3%, *P* < 0.05) than females (n=214, 25%), but the severe root resorption distribution with degree 2‒3 was not significantly different between genders [(133 females (15.5%) and 68 males (13.7%)] (Table 2). The total treatment duration of male patients was significantly longer than in females (Table 3).

**Table 2:** Total and fixed orthodontic treatment durations of male and female patients

**Gender**	**No. of patients**	**Total treatment duration (months, mean ± SD)**	***P*** ** value**	**Fixed orthodontic treatment duration (months, mean ± SD)**	***P*** ** value**
Male	498	28.6±10	0.009*	22.8±9.1	0.063
Female	858	27.1±9.2		23.1±9	

*P < 0.05, one-way ANOVA.

**Table 3:** Analysis of variables related to OIIEARR

	**Orthodontically induced inflammatory external apical root resorption (OIIEARR)**					
	**None** **(degree 0)**	**Resorption (degree 1-2-3)**	***P*** ** value**	**None to mild resorption (degree 0-1)**	**Severe resorption** **(degree 2-3)**	***P*** ** value**
n	981	375		1155	201	
Pre-treatment age (years; mean ± SD)	14.6±2.8	14.3±2.5	0.437^a^	14.4±2.8	14.5±2.6	0.569^a^
Total treatment duration (months)	26.7±9.4	30.3±9.4	0.000^b*^	27.5±9.5	28.7±9.3	0.121^b^
Fixed orthodontic treatment duration (months)	21.9±8.6	25.8±9.6	0.000^b*^	22.9±0.9	23.89.0	0.198^b^
(treatment type) Two-phase treatment (n=455)	n=335 73.6%	n=120 26.4%	0.191^c^	n=387 85.1%	n=68 14.9%	0.372^c^
(treatment type) One-phase treatment with fixed appliances (n=790)	n=562 71.1%	n=228 28.9%	0.191^c^	n=665 84.2%	n=125 15.8%	0.372^c^
Extraction (n=387)	n=229 59.2%	n=158 40.8%	0.000^c*^	n=329 85%	n=58 15%	0.000^c*^
Non-extraction (n=969)	n=751 77.5%	n=218 22.5%	0.000^c*^	n=826 85.2%	n=143 14.8%	0.000^c*^

^a^Independent *t* test, ^b^One-way ANOVA, ^c^Chi-square test, *P < 0.05.

Table 3 presents the relationship between OIIEARR and different variables. There were no significant differences regarding the pre-treatment age and type of orthodontic treatment (two phases or one phase). The total treatment duration of patients developing OIIEARR of any degree was significantly longer, and patients treated with tooth extraction had a greater probability of developing OIIEARR.

Table 4 presents the distribution of the different degrees of OIIEARR for each tooth. OIIEARR incidence rates were significantly higher in the maxillary teeth (33%) than mandibular teeth (16%). Incisors developed a significantly higher incidence of OIIEARR in both the maxilla and mandible. The tooth with the highest incidence of severe OIIEARR with degree 3 was tooth #11 in the maxilla and #46 in the mandible.

**Table 4:** Number and percentage of each tooth presenting different degrees of OIIEARR

**Tooth**	**Degree of OIIEARR**								**Total**
	**Degree 0**		**Degree 1**		**Degree 2**		**Degree 3**		
	**No.**	**%**	**No.**	**%**	**No.**	**%**	**No.**	**%**	
16	1345	99,2%	3	0,2%	4	0,3%	4	0,3%	11
15	1342	99,0%	2	0,1%	8	0,6%	4	0,3%	14
14	1344	99,1%	3	0,2%	7	0,5%	2	0,1%	12
13	1325	97,7%	9	0,7%	20	1,5%	2	0,1%	31
12	1147	84,6%	111	8,2%	88	6,5%	10	0,7%	209
11	1167	86,1%	107	7,9%	70	5,2%	12	0,9%	189
21	1171	86,4%	102	7,5%	74	5,5%	9	0,7%	185
22	1160	85,5%	100	7,4%	90	6,6%	6	0,4%	196
23	1329	98,0%	9	0,7%	16	1,2%	2	0,1%	27
24	1347	99,3%	3	0,2%	5	0,4%	1	0,1%	9
25	1341	98,9%	5	0,4%	7	0,5%	3	0,2%	15
26	1348	99,4%	0	0,0%	4	0,3%	4	0,3%	8
36	1340	98,8%	3	0,2%	10	0,7%	3	0,2%	16
35	1345	99,2%	4	0,3%	6	0,4%	1	0,1%	11
34	1344	99,1%	4	0,3%	6	0,4%	2	0,1%	12
33	1340	98,8%	8	0,6%	7	0,5%	1	0,1%	16
32	1232	90,9%	96	7,1%	27	2,0%	1	0,1%	124
31	1191	87,8%	127	9,4%	36	2,7%	2	0,1%	165
41	1184	87,3%	136	10,0%	34	2,5%	2	0,1%	172
42	1219	89,9%	110	8,1%	26	1,9%	1	0,1%	137
43	1335	98,5%	10	0,7%	10	0,7%	1	0,1%	21
44	1337	98,6%	5	0,4%	12	0,9%	2	0,1%	19
45	1345	99,2%	8	0,6%	3	0,2%	0	0,0%	11
46	1325	97,7%	4	0,3%	19	1,4%	8	0,6%	31

n: number of patients; %: percentage of each tooth presenting different degrees of OIIEARR.

## Discussion

Since external apical root resorption was first reported as an unfavorable side effect of orthodontic treatment by Ottolengui in 1914,^16^ several studies and reviews on this issue have been published. Factors such as orthodontic treatment type,^12^ orthodontic force type and magnitude,^17,18^ treatment duration,^3^ pre-treatment patient age,^4^ and gender^5^ have been associated with OIIEARR. However, only limited information is available in the literature regarding the prevalence and risk factors of OIIEARR. This retrospective study evaluated the incidence, severity, and possible patient- or treatment-related risk factors of OIIEARR in a large sample of orthodontically treated patients. It is important to emphasize the difficulty in finding a sample as large as that of the present study, which is expected to yield more reliable results.

Panoramic radiographs, which had been routinely taken before and after orthodontic treatments, were used in this study to assess root resorption. This might be considered a limitation for the methodology of the study. Extraoral radiographs might be less accurate than other imaging procedures, such as periapical radiographs or 3D images on CT scans to evaluate root resorption.^3,12^ Sameshima and Asgarifar^3^ reported that the amount of root resorption would be exaggerated by 20% or more if the pre- and post-treatment panoramic radiographs were used instead of periapical radiographs. However, many researchers have concluded that a well-taken panoramic film can be as diagnostic as a set of periapical films and can be used to evaluate root resorption with less radiation and better cost-benefit relationships compared with other techniques.^9,11-13,19,20^ In this study, the degree of root resorption was assessed using an ordinal scale in standardized, high-quality panoramic radiographs, comparing the pre- and post-treatment root lengths rather than direct measurements of the absolute values of apical root loss.

External apical root resorption can be observed idiopathically or due to different etiologic factors regardless of the orthodontic treatment.^13^ Some researchers have reported that 7% to 13% of individuals with no orthodontic treatment show apical root resorption to different degrees.^21^ In the present study, the percentage of pre-treatment resorption was 0.04% (8 of 1678). It was decided to exclude these patients from the study because these low numbers would make the pre- and post-treatment adjustments impossible.

In the present study, 27.7% of orthodontically treated patients exhibited OIIEARR of different degrees, and the incidence of severe resorption (with degrees 2‒3) was 14.8%, regardless of the treatment or patient-related variables. Severe root resorption during orthodontic treatment has been reported to occur very rarely, i.e., in 1-5% of patients.^1,10^ The highest incidence of root resorption in the literature was reported by DeShields,^22^ who found root resorption in 99.08% of patients. These differences in the findings are likely due to different variables or assessment techniques.

Some researchers have stated that patient age could be a risk factor for OIIEAR, and orthodontic treatment should be started as young as possible; moreover, adult patients should be informed about this risk.^3,6,23,24^ Pastro et al reported that increased OIIEAR risk in adult patients is associated with an increased incidence of chronic periodontal diseases.^9^ Many studies, with a few exceptions, have found no relationship between OIIEAR and chronological age, as the present study.^2,9-12^

Most studies have found an inconsistent association between gender and OIIEARR.^3,25^ Levander et al^4^ and Kjaer^5^ found a greater prevalence of OIIEARR in females than males. In contrast, Baumrid et al^26^ reported a higher prevalence of OIIEARR in males, consistent with the present study. Jung & Cho,^27^ Pastro et al,^9^ and McFadden et al^28^ found no relationship between gender and OIIEARR prevalence. However, OIIEARR was found to be more frequent in males than females in the present study, and the total treatment duration of the male patients was also longer. The longer total treatment duration in male patients compared to female patients could be associated with a longer pubertal period and less compliance in young males.^27^

The duration of orthodontic treatment has been suggested to contribute significantly to apical root resorption.^4,9,11,24,28,29^ In the present study, the finding that patients who had a longer total treatment duration demonstrated significantly more OIIEARR is consistent with earlier findings. Martins et al^30^ reported that the main factor behind the association between OIIEARR and treatment duration is greater tooth movement. Accordingly, the incidence of OIIEAR was significantly higher in the cases treated with extraction in the present study. This finding is consistent with previous studies.^31^ Beck et al^32^ and Janson et al^23^ reported that extraction treatments are more likely to cause OIIEARR because of the retraction mechanisms of the anterior teeth, causing greater movement of the root apexes and requiring a longer treatment time.

Two-phase orthodontic treatment procedures, i.e., one administered during adolescence and the other administered later during adulthood, have been reported to decrease the extent of OIIEARR.^2,29^ Brin et al^29^ reported a significant increase in the incidence of OIIEARR in patients who were treated with only fixed appliances compared to two-phase treatment. In fact, Brezniak and Wasserstein^2^ maintained that early treatment followed by a second phase of treatment could serve as a protective factor limiting OIIEARR. In the present study, there were no significant differences between two-phase and one-phase treatment protocols regarding the incidence of OIIEARR.

Consistent with the results of other studies,^3,10,11,27^ of all the tooth groups, the maxillary incisors were more likely to exhibit OIIEARR in the present study. The cortical bone of the socket, the proximity between the roots of maxillary central and lateral incisors, the alveolar bone on the buccal surface, the incisive canal, and intrusion and retraction movements are thought to be responsible for the high resorption potential of these teeth.^10,11,33^ Mandibular incisors were found to exhibit OIIEARR after maxillary incisors in the present study. McFadden et al^28^ reported that mandibular incisors are more likely to undergo root resorption after intrusion movement than the maxillary incisors. On the other hand, if the extraction space is used to resolve tooth crowding, which is common for the mandibular arch, incisors might not be subjected to major movements.^1^

## Conclusion

The incidence of severe root resorption after orthodontic treatment was 14.8% in the present study. Significantly related risk factors were prolonged treatment duration and treatment with extraction. However, OIIEARR is a multifactorial phenomenon; therefore, radiographic control should be carried out routinely, especially in patients with orthodontic treatment exceeding six months. Panoramic radiographs can be used to evaluate OIIEARR.

## Authors’ Contributions

FB and EB contributed to the design of the study, data collection, interpretation of the results, editing the manuscript. EB designed the study, interpreted the results, and wrote and edited the manuscript

## Funding

This research did not receive any specific grant from funding agencies in the public, commercial, or not-for-profit sectors.

## Competing Interests

The authors declare no competing interests with regards to the authorship and/or publication of this article.

## Ethics Approval

The study protocol was approved by the Akdeniz University Medical Faculty Ethics Committee before the study (App no: 669).
